# Aerosol Generation During Otologic Surgery

**DOI:** 10.1097/MAO.0000000000003591

**Published:** 2022-07-28

**Authors:** Mari Lahelma, Lotta Oksanen, Noora Rantanen, Saku Sinkkonen, Antti Aarnisalo, Ahmed Geneid, Enni Sanmark

**Affiliations:** ∗ Faculty of Medicine, University of Helsinki; † Department of Otorhinolaryngology and Phoniatrics–Head and Neck Surgery, Helsinki University Hospital; ‡ Faculty of Science, Mathematics, and Statistics, University of Helsinki, Helsinki, Finland

**Keywords:** Aerosol, Aerosol generation, Airborne transmission, COVID-19, Drilling, Otologic surgery

## Abstract

To assess whether aerosol generation occurs during otologic surgery, to define which instruments are aerosol generating, and to identify factors that enhance safety in protection against airborne pathogens, such as severe acute respiratory syndrome coronavirus 2.

**Study Design:**

An observational prospective study on aerosol measurements during otologic operations recorded between August and December 2020.

**Setting:**

Aerosol generation was measured with an Optical Particle Sizer as part of otologic operations with anesthesia. Particles with a size range of 0.3 to 10 μm were quantified. Aerosol generation was measured during otologic operations to analyze aerosols during drilling in transcanal and transmastoid operations and when using the following instruments: bipolar electrocautery, laser, suction, and cold instruments. Coughing is known to produce significant concentration of aerosols and is commonly used as a reference for high-risk aerosol generation. Thus, the operating room background concentration and coughing were chosen as reference values.

**Patients:**

Thirteen otologic operations were included. The average drilling time per surgery was 27.00 minutes (range, 2.00–71.80 min).

**Intervention:**

Different rotation speeds during drilling and other instruments were used.

**Main Outcome Measures:**

Aerosol concentrations during operations were recorded and compared with background and cough aerosol concentrations.

**Results:**

Total aerosol concentrations during drilling were significantly higher than background (*p* < 0.0001, *d* = 2.02) or coughing (*p* < 0.0001, *d* = 0.50). A higher drilling rotation speed was associated with higher particle concentration (*p* = 0.037, *η*^2^ = 0.01). Aerosol generation during bipolar electrocautery, drilling, and laser was significantly higher than with cold instruments or suction (*p* < 0.0001, *η*^2^ = 0.04).

**Conclusion:**

High aerosol generation is observed during otologic surgery when drill, laser, or bipolar electrocautery is used. Aerosol generation can be reduced by using cold instruments instead of electric and keeping the suction on during aerosol-generating procedures. If drilling is required, lower rotation speeds are recommended. These measures may help reduce the spread of airborne pathogens during otologic surgery.

## INTRODUCTION

Coronavirus disease 2019 (COVID-19) is an easily contagious disease and has significantly affected elective otologic procedures; operations have been postponed, waiting times for operations have significantly increased, surgical personnel have been minimized, and the necessary personal protective equipment have been discussed (1,2).

Severe acute respiratory syndrome coronavirus 2 (SARS-CoV-2) can spread through contact, fomites, and airborne routes (3). Airborne transmission is a hypernym for the transmission route containing the continuum from droplets to smaller aerosol particles. Classically, droplets are defined as greater than 5 μm and likely to settle on a surface usually 0.5 to 3 m away from the source, allowing spread only in close contact (4–6). On the other hand, particles less than 5 μm are defined as aerosols. Small aerosol particles persist in the air for long periods of time, and if environmental conditions are optimal, aerosols can move and expose people over longer distances. In addition to effective spread of especially small particles, aerosols have been observed to contain pathogens (7). Accordingly, small (<5 μm) particles are the most challenging for infection control measures (8). In surgery, a particular risk of aerosol generation is associated with the use of electronic devices, such as electrocautery and drilling (1).

Otologic drilling is generally considered as a high-risk procedure for aerosol-generating procedure (AGP) (9). The perception is based on the facts that aerosol generation has been observed during drilling in cadaveric simulations and SARS-CoV-2 has been detected from middle ear secretions (10–12). Detection of SARS-CoV-2 in middle ear secretions is not surprising, as other respiratory viruses in middle ear effusion have been shown to have a high viral concordance (82–98%) (13). Viruses most probably migrate from the pharynx to the middle ear through the eustachian tube. Thus, the British Society of Otology recommends that if drilling is required in otologic surgery, the rotation speed should be as slow as possible to minimize aerosol production (9). However, no real-life measurements for aerosol generation during otologic drilling exist.

In this study, our primary objectives were to explore whether aerosol generation during general anesthesia otologic surgery occurs and, if so, to determine the particle concentrations and size distribution. Second, we examined whether differences emerge in aerosol generation between transcanal and transmastoid operations and whether aerosol generation is influenced by the rotation speed of the drill. In addition to drilling, we explored other possible risk instruments regarding aerosol generation. An understanding of aerosol generation is necessary to make better decisions concerning the protection of healthcare workers and on which operative activities should proceed during the COVID-19 pandemic.

## MATERIALS AND METHODS

### Study Design

Otologic operations and particle measurements were conducted at Helsinki University Hospital, Department of Otorhinolaryngology and Phoniatrics–Head and Neck Surgery from August to December 2020. Measurements were performed as part of normal general or local anesthesia in the operating room (OR) environment without any extra arrangements. Aerosol sampling was continuous throughout the surgery and was conducted with an Optical Particle Sizer (OPS). The OPS was situated, on average, 108 ± 33 cm from the patient's operated ear, vertically at the ear level, and positioned toward the operated ear to reflect the aerosol dose to which the surgeon and assisting nurse are exposed during the surgery. The device was always as close to the patient as possible in the treatment situation and, in principle, was not moved during the procedure. The research nurse followed the surgery and kept a detailed record of all events that occurred during the procedure. Particle concentration was measured continuously throughout the procedure. In the analysis phase, the particle concentration during the use of each individual instrument (e.g., drill) was separately analyzed based on the research nurse's log book.

### Otologic Operations

Drilling was performed using a Stryker Core drill console (Stryker Corp., Kalamazoo, MI) with the following three different handpieces: Stryker Saber Straight Portman Chuck when drilling 15,000 revolutions per minute (rpm), Stryker Saber drill when drilling 60,000 rpm, and Stryker S2 πDrive drill 75,000 RPM when drilling 75,000 rpm. Rotation speed varied from 15,000 to 75,000 rpm. Rotation speed of 15,000 rpm was only used at the end of cochlear implantation operations when drilling the round window niche. Otherwise, surgeons used maximum speed (60,000 or 75,000 rpm, depending on which handpiece was in use) throughout the surgery. The operations were categorized based on location to either transcanal (myringoplasty, stapedotomy) or transmastoid (tympanomastoidectomy, cochlear implantation). The investigated instruments were selected based on general incidence during otologic surgery and previous evidence supporting the potential of aerosol generation (1,9). In addition to drilling, the instruments applied were as follows: cold instruments, suction (Medela Basic 30; Medela, Baar, Switzerland), laser (Diode Laser FOX 980 nm; A.R.C. Laser GmbH, Nuremberg, Germany), and bipolar electrocautery (Valleylab Force FX; Valleylab, Boulder, CO).

### Optical Particle Sizer

OPS (TSI model 3330 OPS) measures particle concentration and size distributions from 0.3 to 10 μm based on optical light scattering. The size and concentration of the particles were registered in 16 size bins every 10 seconds throughout the surgery. The OPS was factory calibrated before the study, and the 1 L/min flow rate was compared with that of the mass flow meter (TSI model 4143). In addition, the size bins were calibrated with polystyrene latex particles with a refractive index of 1.59 before the study.

The air exchange rate varied in the ORs between 30.23 and 31.95 changes per hour and in the central laminar flow area between 542 and 573 changes per hour. This means that all air was changed at the measurement site every 6 to 7 seconds. Because the measurements were performed with an interval of 10 seconds, the role of possible aerosol accumulation in our data remains minimal. All values were above the American Institute of Architects guidelines of a minimum of 25 exchanges per hour (14). The ORs had a Recair 4C ventilation system (ETS Nord, Tuusula, Finland).

### Background and Coughing Aerosol Measurements

Background aerosol size distribution and concentrations were measured before each surgery in empty ORs. In addition, previous (E Sanmark, N Rantanen, M Lahelma, V Anttila, L Lehtonen, AP Hyvärinen, A Geneid, submitted for publication) data from volitional coughs in a similar OR environment were used as a reference. To estimate the aerosol concentration generated by coughing as reliably as possible, other procedures in the OR should be paused during the measurements. There should also be sufficient time between coughs to allow the concentration to return to background levels. These considerations, the fact that patients were in general anesthesia during operations and the real-life nature of this study, did not allow measurement of coughs in anesthetized otologic patients. Therefore, the reference data were collected separately. Coughing has previously been defined as a reference for “highly aerosol-generating procedures” (AGP) by the World Health Organization (6,15,16).

### Statistical Methods

The size-dependent aerosol concentrations measured with OPS were normalized with respect to the sizing bin widths to range from 0.3 to 10 μm. The volume-weighted particle size distribution and total particle concentrations per cubic centimeter were calculated. The particles were categorized based on the following diameters: <1, 1 to 5, and greater than 5 μm. The geometric mean and geometric standard deviation were chosen as statistical representatives, and parametric tests were used for hypothesis testing with log-transformed data. These were chosen because of infection risk being related to total aerosol exposure (17) and the general nature of aerosol data to disperse lognormally (18,19). Although momentary spikes in aerosol concentration were likely to play a significant role in total aerosol exposure, the contribution of these spikes was assumed to be underestimated with median and nonparametric tests. The observed aerosol concentrations were compared with baseline and coughing references using unpaired Student's two-sided *t* test. Differences in aerosol concentrations between techniques were analyzed using two-way analysis of variance with Tukey HSD post hoc test for multiple comparisons. For each aerosol size category, two models were formed. The first model analyzed effects of drilling rotation speed (1. independent variable) and location (2. independent variable), and the second model analyzed effects of different instruments (independent variable) and location (covariate). Before analysis, the data were log_10_ normalized (17). The analyses were performed with RStudio version 1.3.959 (R Foundation for Statistical Computing, Vienna, Austria) or GraphPad Prism version 9.0.2 for Mac (GraphPad Software, San Diego, CA). A *p* value less than 0.05 was considered as statistically significant.

### Ethical Considerations

All procedures that involved human participants were conducted in accordance with the ethical standards of the institutional or national research committee and the 1964 Declaration of Helsinki and its later amendments or comparable ethical standards. The Ethics Committee of Helsinki University Hospital approved the study protocol (HUS/1701/2020). All patients provided written informed consent before participation.

## RESULTS

### General Characteristics of Patients and operations

**FIG. 1:**
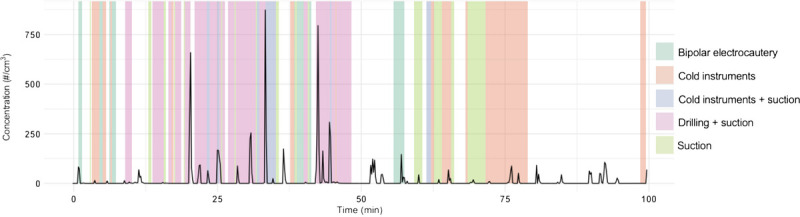
Total particle concentration during an example cochlear implantation surgery. Time periods of drilling, bipolar electrocautery, suction, and cold instruments are indicated with separate colors. Total particle concentration was measured with a 10-second scale interval using an Optical Particle Sizer (TSI model 3330 OPS). Operating room information: temperature of 20.7°C and humidity of 40.90%.

A total of 13 patients and the corresponding otologic operations (8 transcanal and 5 transmastoid) were included in the study. Patient and surgery characteristics are presented in Table 1. The average duration of surgery was 128.46 ± 52.06 minutes (range, 68.00–229.17 min). A total of 10,020 aerosol measurement points (10-s recording per measurement point) was recorded. The lowest observed total aerosol concentration was zero (0.000) particles/cm^3^, and the highest was 1353.132 particles/cm^3^. Zero particles were observed during 11.97% of the measurements. An example timeline of cochlear implantation with momentary aerosol concentrations and instruments utilized is shown in Figure 1.

**TABLE 1:** General characteristics of patients and operations

	Transcanal	Transmastoid	*p*
Patients			
n	8	5	
Sex (female)	4 (50%)	3 (60%)	1.000
Age (yr)	43.4 ± 14.0	50.2 ± 21.0	0.511
Height (cm)	172.6 ± 9.5	168.3 ± 7.1	0.361
Weight (kg)	82.8 ± 11.3	99.1 ± 29.5	0.246
BMI (kg/m^2^)	27.7 ± 1.8	34.8 ± 9.1	0.115
Operations			
Surgery (n)	Myringoplasty (3) Stapedotomy (5)	Cochlear implantation (2) Tympanomastoidectomy (3)	
Duration (min)	102.0 ± 29.7	136.4 ± 55.8	0.206
OPS situation (cm from patient's ear)	105.0 ± 37.9	111.7 ± 29.1	0.716
Temperature (°C)	20.5 ± 0.3	20.2 ± 0.2	0.122
Humidity (%)	41.3 ± 14.3	44.6 ± 10.3	0.668

Data are presented as mean ± standard deviation or n (%) as appropriate. Differences between groups were calculated using unpaired Student's two-sided *t* test for continuous variables and Fisher's exact test for categorical variables.

BMI indicates body mass index; OPS, Optical Particle Sizer.

The background aerosol concentrations were 4.2 × 10^−5^ ± 16.333 particles/cm^3^ for total particle concentration and 1.2 × 10^−5^ ± 3.100, 1.0 × 10^−5^ ± 1.459, and 1.1 × 10^−5^ ± 1.919 particles/cm^3^ for particle concentrations of less than 1, 1 to 5, and greater than 5 μm, respectively. For coughing, aerosol concentrations were 0.007 ± 58.931 for total particle concentration and 0.005 ± 58.110, 3.9 × 10^−4^ ± 39.473, and 1.7 × 10^−5^ ± 5.873 particles/cm^3^ for particle concentrations less than 1, 1 to 5, and greater than 5 μm. Cough measurements have been described previously (E Sanmark, N Rantanen, M Lahelma, V Anttila, L Lehtonen, AP Hyvärinen, A Geneid, submitted for publication).

### Aerosol Generation During Otologic Drilling

**FIG. 2:**
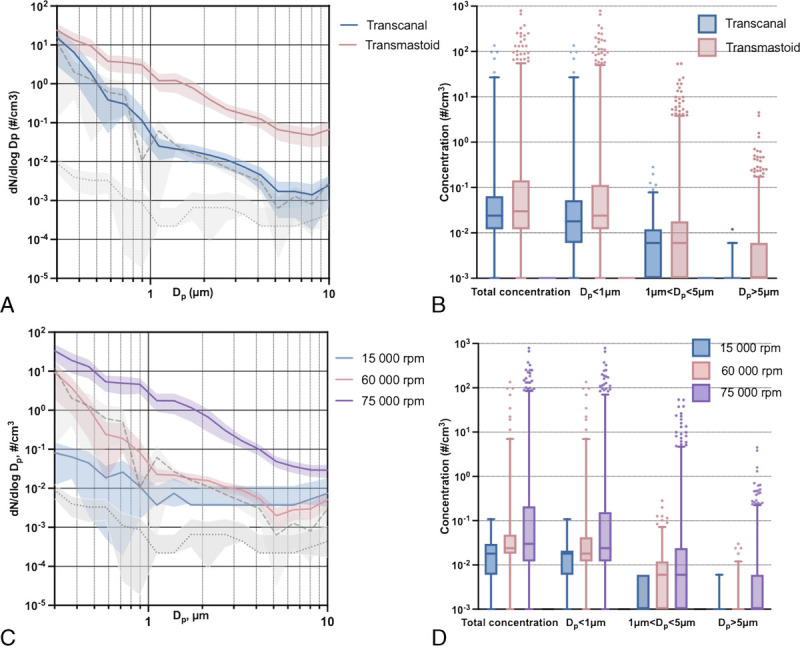
Aerosol generation during otologic drilling. Average size distribution of observed aerosols (*A*) during drilling in transcanal and transmastoid operations and (*C*) with different rotation speeds. Dotted gray line represents observed aerosol size distribution at background and dashed gray line during coughing. Data are presented as mean (line) with 95% confidence intervals (envelopes). Total concentrations and concentrations of diameter particles of less than 1, 1 to 5, and greater than 5 μm observed (*B*) during drilling in the transcanal and transmastoid operations and (*D*) during drilling with different rotation speeds, presented as median with interquartile range (box) and 2.5 to 97.5 percentiles (whiskers). Momentary spikes in aerosol concentrations are seen as several upper outliers (dots). See SDC, Table 1, http://links.lww.com/MAO/B469, for pairwise comparisons between different locations and rotation speeds. Dp refers to diameters of the observed particles, and dN/dlogDp is the concentration expressed as particles per cubic centimeter. Transcanal operations, n = 8; transmastoid operations, n = 5. c indicates concentration; rpm, revolutions per minute.

Drilling was performed in eight (62%) of the operations (three transcanal and five transmastoid). The average drilling time was 27.00 minutes (range, 2.00–71.80 min). The aerosol concentrations and size distribution during drilling are presented in Table 2 and Figure 2, A and B. Drilling in the transcanal operations produced less aerosols than drilling in the transmastoid operations in all size categories when adjusted for drilling rotation speed (Table 2, Fig. 2B; Supplemental Digital Content [SDC] Table 1, http://links.lww.com/MAO/B469).

**TABLE 2:** Aerosol concentrations during otologic drilling and from other instruments

	Total Duration (min)	Total Particle Concentration (No./cm^3^)	<1 μm Particle Concentration (No. /cm^3^)	1–5 μm Particle Concentration (No./cm^3^)	>5 μm Particle Concentration (No./cm^3^)
		Mean ± SD	Mean ± SD	Mean ± SD	Mean ± SD
1 Drilling	215.83	0.037 ± 22.335***	0.026 ± 27.074***	7.9 × 10^−4^ ± 62.187***	5.7 × 10^−5^ ± 62.187***
1.1 Location					
Transcanal	37.33	0.020 ± 23.300***	0.012 ± 32.899***	4.4 × 10^−4^ ± 33.822	2.0 × 10^−5^ ± 7.599
Transmastoid	178.50	0.042 ± 21.849***	0.031 ± 25.460***	9.0 × 10^−4^ ± 69.249***	7.1 × 10^−5^ ± 28.166***
*p^a^*		**0.004**	**0.002**	**0.012**	**0.001**
1.2 Rotation speed (rpm)				
15,000	2.83	0.007 ± 14.257	0.004 ± 21.343	0.7 × 10^−4^ ± 20.174*	3.1 × 10^−5^ ± 12.350
60,000	63.50	0.031 ± 8.093***	0.019 ± 12.308***	7.5 × 10^−4^ ± 30.490*	2.8 × 10^−5^ ± 10.986***
75,000	152.17	0.041 ± 31.41***	0.031 ± 35.579***	8.5 × 10^−4^ ± 81.789***	7.9 × 10^−5^ ± 30.938***
*p^b^*		**0.037**	**0.009**	0.366	**<0.0001**
2 Other instruments					
Cold instruments	447.50	0.007 ± 28.081	0.004 ± 36.945	1.5 × 10^−4^ ± 32.668***	2.0 × 10^−5^ ± 7.76*
Laser	20.83	0.024 ± 18.274***	0.016 ± 28.592**	9.0 × 10^−4^ ± 22.262***	1.7 × 10^−5^ ± 5.711
Bipolar electrocautery	58.00	0.031 ± 14.538***	0.021 ± 19.432***	4.8 × 10^−4^ ± 39.143	3.4 × 10^−5^ ± 12.981***
Suction	269.33	0.008 ± 37.221	0.004 ± 48.073	2.7 × 10^−4^ ± 39.535	2.8 × 10^−5^ ± 11.042***
*p^c^*		**<0.0001**	**<0.0001**	**<0.0001**	**<0.0001**
3 Coughing (reference)		0.007 ± 58.931	0.005 ± 58.110	3.9 × 10^−4^ ± 39.473	1.7 × 10^−5^ ± 5.873

Significant *p*-values are bolded.

Particle concentrations were compared with the background and coughing references using unpaired Student's two-sided *t* test. All comparisons between particle concentrations and background were significant (*p* < 0.0001). Asterisks represent *p* values for the comparison with coughing. The measured aerosol concentrations during coughing are presented at the end of the table. Total duration describes the total particle recording time. Effects of location and surgery instruments on aerosol concentrations were calculated using two-way analysis of variance with Tukey HSD post hoc test for multiple comparisons. For pairwise comparisons of the rotation speeds and instruments, see Supplemental Table 1 and 2, http://links.lww.com/MAO/B469, respectively. The presented mean and standard deviation are geometric measures.

*^a^p* Value for the comparison of aerosol concentration during drilling in transcanal versus transmastoid operations adjusted for drilling rotation speed.

*^b^p* Value for the comparison of aerosol concentration during drilling with different rotation speeds adjusted for surgery location.

*^c^p* Value for the comparison of aerosol concentration during use of different instruments adjusted for surgery location.

*p < 0.05, for *p* value compared with coughing.

**p < 0.01, for *p* value compared with coughing.

***p < 0.001, for *p* value compared with coughing.

rpm indicates revolutions per minute; SD, standard deviation.

### Aerosol Generation With Respect to Drilling Rotation Speed

Drilling was performed using rotation speeds of 15,000, 60,000, and 75,000 rpm, with total recording durations of 2.83, 63.50, and 152.17 minutes, respectively. The particle size distribution and concentrations during drilling by rotation speed are presented in Table 2 and Figure 2, C and D. The observed aerosol concentrations were significantly different between drilling rotation speeds when adjusted for surgery location (transcanal or transmastoid); the highest concentrations were observed at the highest rotation speeds. Pairwise comparisons of particle concentrations during different rotation speeds are shown in SDC Table 1, http://links.lww.com/MAO/B469.

### Aerosol Generation With Other Instrumentation

**FIG. 3:**
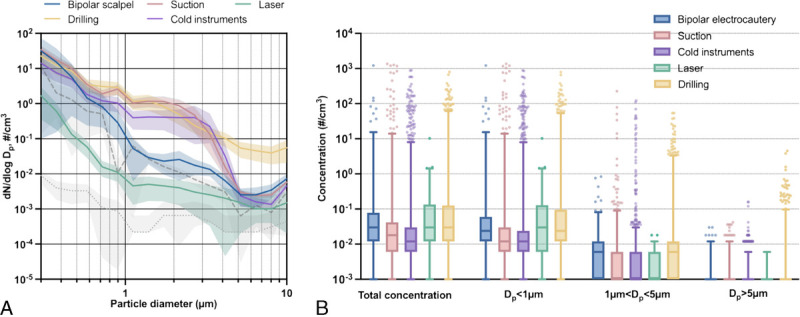
*A*, Average size distribution of observed aerosols during use of different otologic instruments. Dotted gray line represents observed aerosol size distribution at background and dashed gray line during coughing. Data are presented as mean (line) with 95% confidence intervals (envelopes). *B*, Total concentrations and concentrations of aerosols of less than 1, 1 to 5, and greater than 5 μm in diameter observed during different instrument use, presented as median with interquartile range (box) and 2.5 to 97.5 percentiles (whiskers). Momentary spikes in aerosol concentrations are seen as several upper outliers (dots). See SDC, Table 2, http://links.lww.com/MAO/B469, for pairwise comparisons between instruments. Dp refers to diameters of the observed particles, and dN/dlogDp is the concentration expressed as particles per cubic centimeter. *C* indicates concentration; *D*, diameter.

In addition to drilling, aerosol concentrations were measured when using cold instruments, laser, bipolar electrocautery, and suction. The particle size distribution and concentrations observed with these instruments are shown in Table 2 and Figure 3 and compared pairwise with adjustment for surgery location in SDC Table 2, http://links.lww.com/MAO/B469. The highest aerosol concentrations were observed during drilling and bipolar electrocautery; these concentrations were significantly higher than those observed during cold instrument use or mere suction.

### Effect of Suction on Aerosol Generation During Use of Other Instruments

Drilling, cold instruments, laser, and bipolar electrocautery were used either with or without suction (SDC Fig. 1, http://links.lww.com/MAO/B469). Concentrations were lower with suction than without suction in the following particle size subgroups: 1- to 5-μm particles when using cold instruments (−3.888 ± 1.570 versus −3.725 ± 1.497 log_10_(particles/cm^3^); *p* = 0.009; with versus without suction) and bipolar electrocautery (−3.769 ± 1.496 versus −3.181 ± 1.598 log_10_(particles/cm^3^); *p* = 0.003; with versus without suction) and greater than 5-μm particles when using bipolar electrocautery (−4.861 ± 0.609 versus −4.358 ± 1.201 log_10_(particles/cm^3^); *p* < 0.0001; with versus without suction).

## DISCUSSION

This is the first study that measured aerosol generation during different, real-life otologic operations and compared different surgical techniques and equipment with respect to aerosol production. We revealed that drilling in both transmastoid and transcanal operations involves a significant risk of aerosol release. The drilled mucosal area of the tympanomastoid air cell system is connected to the nasopharynx and oropharynx, where high viral loads of respiratory pathogens such as SARS-CoV-2 have been observed (12,13,20). Our findings are important when planning elective surgery and use of personal protective equipment, especially during the COVID-19 pandemic and other epidemics caused by airborne pathogens.

There is no clear limit for significant aerosol production during surgical procedures. However, both the World Health Organization (21) and earlier studies (6,22) have used cough as a reference limit for high-risk AGPs. We have also previously published quantitative measures on aerosol particles generated during coughing in similar OR environments to the otologic operations investigated here (E Sanmark, N Rantanen, M Lahelma, V Anttila, L Lehtonen, AP Hyvärinen, A Geneid, submitted for publication). These measures were used as a reference for AGP in this study. By comparing with the background, we identified whether the procedure generates aerosol particles at all; by comparing the procedure with coughing, we defined the AGPs. Determining a more precise limit would require more information on the required infectious dose, which is not yet available for several respiratory diseases, including COVID-19. Because of the quantitative nature of our data, the results can be utilized to expand knowledge of infectious dose and exposure time in different airborne-transmitted diseases.

We observed higher aerosol concentrations when drilling was performed with maximum rotation speeds than slower rotation ones. This supports the British Society of Otology's recommendation that when drilling is necessary, it should be performed at the slowest possible rotation speed to reduce aerosol generation (9). Similar observations on the role of rotation speed have been made in dental drilling (23). The tissue to be drilled likely influences the generated aerosol concentration. In this study, only bone was drilled, and a significant difference was observed between 60,000 and 75,000 rpm; these speeds were also used when drilling in the exact same location. Thus, the rotational speed would also seem to be an independent factor influencing aerosol generation. In addition, we observed that drilling in transmastoid operations produced more particles than drilling during transcanal operations. However, transcanal drilling also exceeded the particle concentrations observed in the background and during coughing; thus, both procedures can be considered as AGPs. One explanation for the lower concentrations during transcanal operations is the nature of the procedure; the drilling is performed from a smaller opening and deep in the ear canal. In general, in transcanal surgery, small diamond burs are also used. This contrasts with transmastoid surgery, where large cutting burs are preferred at the start of the procedure when working close to the surface. At the later stages of mastoidectomy, diamond burs are also used. As drilling during transcanal middle ear surgery is usually performed after elevation of the tympanomeatal flap and involves drilling of scutum bone, which is in contact with tympanic mucosa, drilling in transcanal middle ear procedures can be regarded as a high-risk AGP.

Time is a significant variable when assessing the likelihood of exposure for infection. For COVID-19, the critical exposure limit has been evaluated to be 15 minutes for contact tracing. However, the role of aerosol accumulation has not been considered separately in these estimates (24). The average drilling durations were 12 minutes in transcanal and 36 minutes in transmastoid operations, with the latter markedly exceeding the time for high-risk bioaerosol exposure, thus increasing the risk for airborne infections like influenza and COVID-19 (25). SARS-CoV-2 has been detected in the middle ear, and significant concentrations of aerosols are generated during ear drilling (12). Moreover, because the exposure times are quite long, personal protective equipment against bioaerosols should be used in otologic operations that include drilling in the middle ear or a perforated ear drum.

We also observed high aerosol concentrations when using bipolar electrocautery and laser. Adding suction decreased aerosol generation. Although previous studies have also reported aerosol generation with powered instruments, the effect of suction is controversial (1,26). Both increased and decreased aerosol concentrations have been reported during suction (27–30). Recently, however, the prevailing view has been that suction reduces aerosol concentration (28,30,31). Our results are consistent with this view, and we recommend applying suction, especially when using powered instruments.

We measured particle size and distribution at the same distances from the patient that the OR staff are typically positioned. Thus, the results reliably reflect the aerosol dose to which the staff is exposed in the OR but not the total particle concentration generated during the surgery. The ventilation conditions in the OR and outpatient facilities are different, and the results cannot be directly applied outside the OR, where, for example, the accumulation of aerosols is likely to be a more significant factor. Because of the real-life environment and the nature of the study, the localization of the OPS device varied between the patients. Although this can be considered as a limitation, measuring the entire procedure with living patients provides a more representative overall picture of the actual aerosol exposure that OR staff encounter during surgery. Aerosols are influenced by various environmental factors also in highly ventilated spaces (such as ORs), which can also affect aerosol concentrations. There was also no control group in the study, because of its real-life nature and the use of real patients as patient material. In this study, all operations were performed by trained otologists and with proper techniques, which may influence aerosol production. Thus, these results may underestimate the aerosol production in less experienced practitioners, for example, in resident training. Breathing has been observed to generate some aerosol and may potentially cause bias in surgery measurements (32). However, in our study, all staff used masks throughout the operations, which has earlier been observed to efficiently reduce aerosol release (33,34). Most of the operations were performed under general anesthesia; the patient's breathing did not impact the results as the exhaled air is filtered. In the operations performed under local anesthesia, the OPS was localized to the patient's occipital side to reduce bias from potential particles generated during patient breathing.

## CONCLUSIONS

Drilling in otologic operations produces significant concentrations of aerosols that can carry airborne pathogens, such as SARS-CoV-2 and influenza. Considering the long duration of drilling, especially during transmastoid operations, transmastoid otologic surgery that includes drilling can be considered a high-risk AGP. Transcanal operations can also be regarded as high-risk AGPs if drilling involves bone that is attached to tympanic mucosa. Aerosol generation during otologic surgery can be reduced by using lower rotation speeds when drilling, choosing cold instruments over electric ones, and maintaining suction during AGPs.

## Supplementary Material
