# Supplementary material for: Aerosol Generation During Otologic Surgery
Source: Otol Neurotol. 2022 Jul 28;43(8):924–30. doi: 10.1097/MAO.0000000000003591 (PMC9394486; doi:10.1097/MAO.0000000000003591)
Supplement: Supplementary file 1 [file on-43-0924-s001.docx]

**SDC Table 1.** Pairwise comparisons of aerosol concentrations observed during drilling between different locations and rotation speeds.

|  | Total particles | | <1 𝜇m particles | | 1–5 𝜇m particles | | >5 𝜇m particles | |
| --- | --- | --- | --- | --- | --- | --- | --- | --- |
|  | Difference  (95% CI) | p-value | Difference  (95% CI) | p-value | Difference (95% CI) | p-value | Difference (95% CI) | p-value |
| ***Location*** |  |  |  |  |  |  |  |  |
| Transmastoid – Transcanal | 0.26 (-0.07 – 0.46) | **0.004** | 0.29 (0.09 – 0.50) | **0.002** | 0.18 (-0.25 – 0.61) | 0.012 | 0.30 (0.11 – 0.50) | **0.001** |
| ***Rotation speed*** |  |  |  |  |  |  |  |  |
| 60 000 – 15 000 | 0.60 (-0.18 – 1.38) | 0.173 | 0.61 (-0.22 – 1.44) | 0.198 | 0.00 (-1.75 – 1.76) | 1.000 | -0.04 (-0.83 – 0.74) | 0.992 |
| 75 000 – 15 000 | 0.72 (-0.05 – 1.50) | 0.074 | 0.80 (-0.01 – 1.63) | 0.058 | 0.56 (-1.17 – 2.30) | 0.726 | 0.39 (-0.38 – 1.17) | 0.465 |
| 75 000 – 60 000 | 0.12 (-0.07 – 0.31) | 0.292 | 0.19 (-0.01 – 0.39) | 0.071 | 0.55 (0.12 – 0.98) | **0.007** | 0.43 (0.24 – 0.62) | **<0.0001** |

Results from two-way ANOVA post-hoc Tukey HSD test for multiple pairwise comparisons. Difference describes the difference between the means (log_10_) of the groups followed by the 95% confidence interval. Differences between transmastoid (n=5) and transcanal (n=8) surgeries are adjusted by drilling rotation speed and differences between drilling rotation speed by drilling location. Calculations were performed with RStudio version 1.3.959 (R Foundation for Statistical Computing, Vienna, Austria) for log_10_-transformed particle concentrations. CI, confidence interval.

**SDC Table 2.** Pairwise comparisons of aerosol concentrations observed between different locations and instruments used.

|  | Total particles | | <1 𝜇m particles | | 1–5 𝜇m particles | | >5 𝜇m particles | |
| --- | --- | --- | --- | --- | --- | --- | --- | --- |
|  | Difference  (95% CI) | p-value | Difference  (95% CI) | p-value | Difference (95% CI) | p-value | Difference (95% CI) | p-value |
| ***Location*** |  |  |  |  |  |  |  |  |
| Transmastoid – Transcanal | -0.01 (-0.11 – 0.08) | 0.775 | -0.05 (-0.15 – 0.05) | 0.350 | 0.20 (0.09 – 0.30) | **<0.001** | 0.14 (0.07 – 0.21) | <0.001 |
| ***Instrument*** |  |  |  |  |  |  |  |  |
| Suction - Bipolar scalpel | -0.56 (-0.8 – -0.32) | **<0.001** | -0.65 (-0.91 – -0.39) | **<0.001** | -0.25 (-0.52 – 0.01) | **0.070** | -0.08 (-0.25 – 0.09) | 0.735 |
| Cold instruments - Bipolar scalpel | -0.62 (-0.84 – -0.39) | **<0.001** | -0.71 (-0.95 – -0.47) | **<0.001** | -0.47 (-0.72 – -0.22) | **<0.001** | -0.2 (-0.37 – -0.04) | **0.006** |
| Laser - Bipolar scalpel | -0.10 (-0.51 – 0.30) | 0.954 | -0.12 (-0.56 – 0.32) | 0.945 | -0.73 (-1.18 – -0.27) | **<0.001** | -0.3 (-0.60 – 0.00) | 0.050 |
| Drilling - Bipolar scalpel | 0.07 (-0.16 – 0.31) | 0.904 | 0.09 (-0.16 – 0.34) | 0.870 | 0.21 (-0.04 – 0.48) | 0.171 | 0.23 (0.05 – 0.40) | **0.003** |
| Cold instruments - Suction | -0.05 (-0.19 – 0.07) | 0.773 | -0.06 (-0.21 – 0.08) | 0.764 | -0.21 (-0.37 – -0.06) | **0.001** | -0.12 (-0.22 – -0.02) | 0.006 |
| Laser - Suction | 0.45 (0.08 – 0.82) | **0.006** | 0.52 (0.12 – 0.92) | **0.003** | -0.47 (-0.88 – -0.06) | **0.014** | -0.22 (-0.49 – 0.04) | 0.168 |
| Drilling - Suction | 0.64 (0.48 – 0.79) | **<0.001** | 0.74 (0.56 – 0.91) | **<0.001** | 0.47 (0.29 – 0.64) | **<0.001** | 0.31 (0.19 – 0.42) | **<0.001** |
| Laser - Cold instruments | 0.51 (0.15 – 0.87) | **<0.001** | 0.59 (0.2 – 0.97) | **<0.001** | -0.25 (-0.65 – 0.14) | 0.396 | -0.09 (-0.36 – 0.17) | 0.861 |
| Drilling - Cold instruments | 0.69 (0.56 – 0.83) | **<0.001** | 0.80 (0.66 – 0.94) | **<0.001** | 0.68 (0.53 – 0.83) | **<0.001** | 0.44 (0.34 – 0.53) | **<0.001** |
| Drilling - Laser | 0.18 (-0.18 – 0.55) | 0.654 | 0.21 (-0.18 – 0.6) | 0.587 | 0.94 (0.53 – 1.35) | **<0.001** | 0.53 (0.26 – 0.80) | **<0.001** |

Results from two-way ANOVA post-hoc Tukey HSD test for multiple pairwise comparisons. Difference describes the difference between the means (log_10_) of the groups followed by the 95% confidence interval. Differences between transmastoid (n=5) and transcanal (n=8) surgeries are adjusted by used instruments and differences between used instruments speed by location of surgery. Calculations were performed with RStudio version 1.3.959 (R Foundation for Statistical Computing, Vienna, Austria) for log_10_-transformed particle concentrations. CI, confidence interval.
