# Supplementary figures and images for: Aerosol Generation During Otologic Surgery
Source: Otol Neurotol. 2022 Jul 28;43(8):924–30. doi: 10.1097/MAO.0000000000003591 (PMC9394486; doi:10.1097/MAO.0000000000003591)

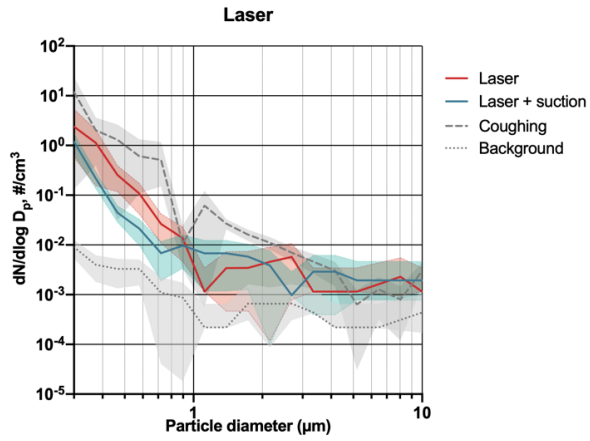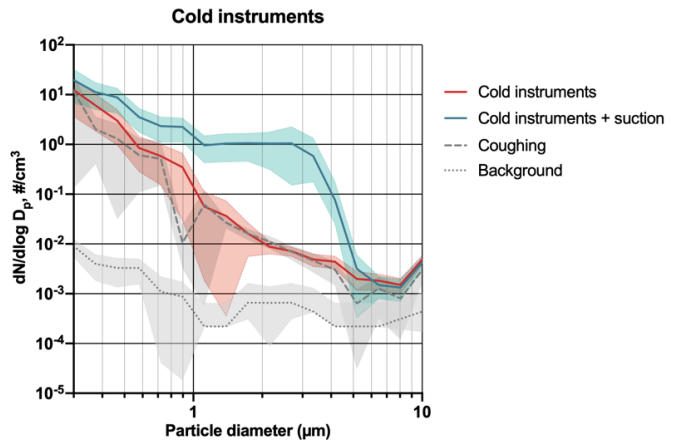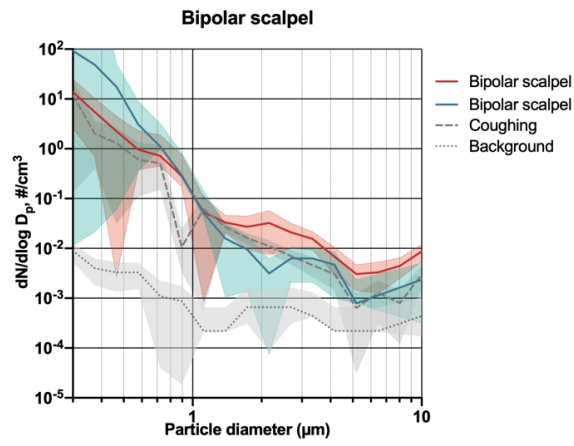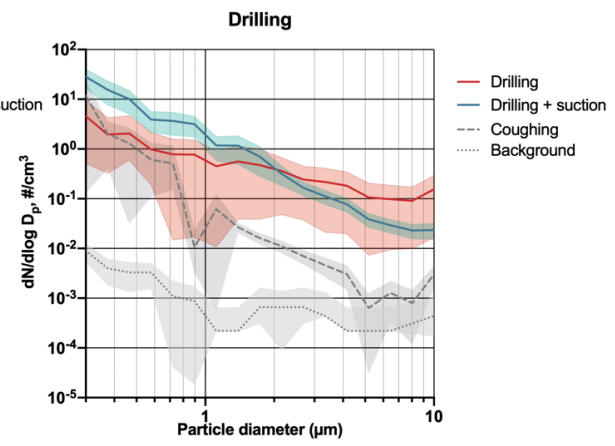

Supplement: Supplementary file 2 [file on-43-0924-s002.pdf]
